# Modulation of Monocyte Effector Functions and Gene Expression by Human Cytomegalovirus Infection

**DOI:** 10.3390/v16121809

**Published:** 2024-11-21

**Authors:** Matthew S. Planchon, Jay A. Fishman, Joseph El Khoury

**Affiliations:** 1 Center for Immunology and Inflammatory Diseases, Massachusetts General Hospital and Harvard Medical School, Boston, MA 02129, USA; matthew.planchon@students.jefferson.edu; 2 Sidney Kimmel Medical College at Thomas Jefferson University, Philadelphia, PA 19107, USA; 3 Transplant Infectious Disease and Compromised Host Program, Division of Infectious Diseases, Massachusetts General Hospital, and Harvard Medical School, Boston, MA 02114, USA; jfishman@mgh.harvard.edu

**Keywords:** cytomegalovirus, monocyte, phagocytosis, chemotaxis, migration, cytokine production, reactive oxygen species, tissue repair, antigen presentation, immunocompromised

## Abstract

Monocytes are crucial players in innate immunity. The human cytomegalovirus (CMV) infection has significant impacts on monocyte effector functions and gene expression. CMV, a β-herpesvirus, disrupts key monocyte roles, including phagocytosis, antigen presentation, cytokine production, and migration, impairing their ability to combat pathogens and activate adaptive immune responses. CMV modulates monocyte gene expression, decreasing their capacity for antigen presentation and phagocytosis while increasing pro-inflammatory cytokine production, which can contribute to tissue damage and chronic inflammation. CMV also alters monocyte migration to sites of infection while promoting trans-endothelial migration, thus aiding viral dissemination. Additionally, the virus affects reactive oxygen species (ROS) production, thereby contributing to end-organ disease associated with CMV infection. Overall, these changes enhance viral persistence during acute infection and facilitate immune evasion during latency. We highlight the clinical significance of these disruptions, particularly in immunocompromised patients such as transplant recipients, where the modulation of monocyte function by CMV exacerbates risks for infection, inflammation, and graft rejection. An understanding of these mechanisms will inform therapeutic strategies to mitigate CMV-related complications in vulnerable populations.

## 1. Introduction

Human cytomegalovirus (CMV) is a ubiquitous DNA β-herpesvirus found worldwide. The global seroprevalence of CMV is estimated to be 56–94%, with differences dependent on country of origin and socio-economic status of infected individuals [1]. CMV infects many cell types, including epithelial cells in the oropharynx, vascular endothelial cells, hepatocytes, fibroblasts, smooth muscle cells, monocytes, macrophages, and dendritic cells. CMV enters the cell nucleus as an episomal element. Like other herpesviruses, CMV establishes lifelong infection, including latent infection in myeloid cells of the bone marrow, where the outcome of infection depends on the cell type [2,3]. Virally encoded proteins from virions or expressed in the cell during active infection, latency, or reactivation, control cellular functions necessary for further viral replication and spread [4]. For example, CMV generally causes lytic infection in fibroblasts, while in myeloid progenitor cells, the initial burst of lytic gene expression is followed by viral latency, allowing infected monocytes to spread the virus systemically. In immunocompetent individuals, the host immune response controls acute CMV infection, which is generally limited to asymptomatic or mild mononucleosis-like symptoms. In the absence of a fully functioning immune system, as in individuals following solid organ or bone marrow transplant (SOT or BMT) or with uncontrolled HIV infection, acute CMV infection or the reactivation of latent virus can be associated with viral replication leading to end-organ disease (EOD) and may contribute to rejection of transplanted organs [5]. Additionally, primary CMV infection during pregnancy poses a risk for congenital birth defects including neonatal hearing loss, microcephaly, and other neurodevelopmental conditions [6].

Monocytes and macrophages are major components of the innate immune system and are important targets for primary CMV infection and reactivation [7]. Here we focus on reviewing the changes in gene expression and cell function associated with CMV infection/reactivation in monocytes and macrophages and highlighting the importance of these changes in the setting of SOT recipients.

## 2. Monocyte Functions

**Figure 1:**
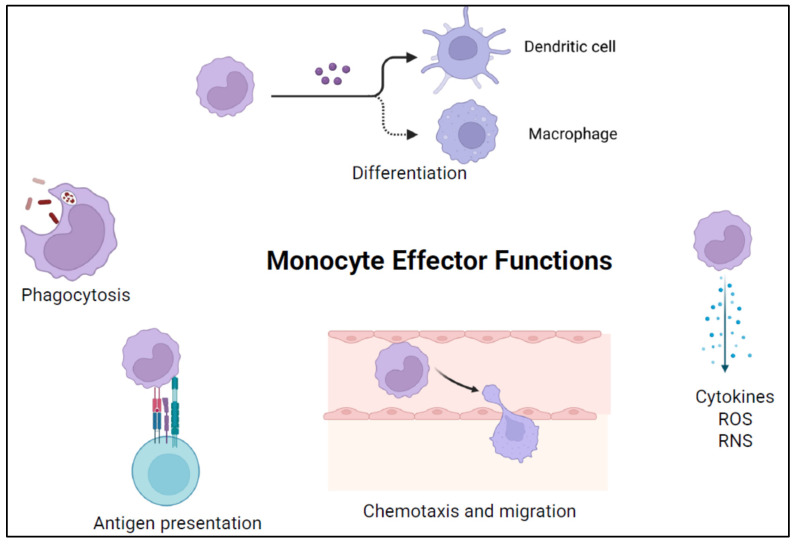
Monocyte effector functions.

Monocytes are bone marrow-derived circulating immune cells responsible for many innate immune responses. There are three major subsets of human monocytes classified based on the relative expression of the cell surface markers CD14 and CD16: classical (CD14++, CD16−), intermediate (CD14+, CD16+), and nonclassical (CD14+, CD16++) monocytes [8]. Additionally, monocyte cell subsets are differentiated based on function (Figure 1). Classical monocytes are the most prevalent subtype and are predominantly responsible for phagocytosis and antigen presentation. Intermediate monocytes are the next most prevalent subtype, and mediate inflammation through the generation of reactive oxygen and nitrogen species (ROS and RNS), antigen presentation for adaptive immune responses, and T cell activation. Non-classical monocytes share similar functions with intermediate monocytes; these include secreting cytokines, activating T cells, and functioning in antigen presentation [9,10]. A potential fourth subset has been described in the literature based on single cell RNASeq data [11]. This subset combines parts of classical monocyte gene expression with a cytotoxic killing profile dictated by perforin 1 (PRF1), granulysin (GNLY), and cathepsin W (CTSW) expression. This subset of monocytes with significant cytotoxic killing ability is not yet well described in the literature and could contribute to monocyte adaptive immunity.

Monocytes are crucial effectors in infection. In acute infections, monocytes migrate into tissues where they can differentiate into macrophages and dendritic cells [9]. Additionally, the vascular and neural accumulation of monocytes plays a role in the pathogenesis of many chronic and inflammatory diseases including atherosclerosis, Alzheimer’s disease, and multiple sclerosis [12,13,14]. Monocytes are crucial in CMV pathogenesis in part due to their differentiation into macrophages within tissues. The lifespan of monocytes in circulation is short at approximately three weeks. However, differentiation into macrophages increases their life span to years [15]. The longevity of macrophages provides a stable reservoir for CMV persistence in multiple tissues and is responsible for clinical latency [1].

Monocyte polarization in response to a variety of stimuli has been extensively studied [16]. A commonly used classification is the M1/M2 classification. While this classification has been useful, subsequent research showed that it is too simplistic and does not reflect the various polarized states of monocytes and macrophages [17].

Monocytes are versatile immune cells that perform several critical functions in the immune response (Figure 1). These functions include:

A. *Phagocytosis:* Monocytes can engulf and digest self- and non-self particles including pathogens and debris.
B. *Antigen Presentation:* Monocytes directly present antigens to T cells and can differentiate into macrophages and dendritic cells which are antigen-presenting cells, initiating adaptive immune responses.
C. *Cytokine Production:* Monocytes produce cytokines and chemokines that modulate the immune response.
D. *Chemotaxis:* Monocytes migrate to sites of infection or injury in response to chemokine signals.
E. *Tissue Repair:* Monocyte-derived macrophages and dendritic cells have specialized functions in tissue repair and immune regulation.
F. *ROS and RNS Production:* Monocytes produce ROS and RNS involved in host defense and tissue damage.

### 2.1. Phagocytosis

Monocytes are among the first responders to infection or tissue damage. Phagocytosis, the ingestion and destruction of invading pathogens such as bacteria, fungi and virus infected cells, as well as apoptotic cells and cellular debris, is a fundamental function of monocytes [18,19,20]. This action helps clear infections and initiates the process of healing and tissue repair. The engulfment of pathogens serves as a precursor to antigen presentation, a crucial step in activating the adaptive immune response. Monocytes recognize pathogens through pattern recognition receptors (PRRs) such as scavenger and toll-like receptors (TLR) [21,22], Fc receptors [23], and complement receptors [24], and initiate phagocytosis. Once a particle is recognized and the phagocytic process is initiated, a specialized vacuole, called phagosome, forms, which matures into a phagolysosome [25].

### 2.2. Antigen Presentation

After phagocytosis, monocytes act as antigen-presenting cells (APCs), processing pathogens and presenting their antigens on the monocyte cell surface. Monocytes express major histocompatibility complex (MHC) class II molecules, which are essential for presenting exogenous antigens to CD4+ T helper cells. Additionally, monocytes can cross-present antigens on MHC class I molecules to CD8+ cytotoxic T cells, a function that is critical for antiviral immunity [26]. By presenting antigens, monocytes help bridge the innate and adaptive immune systems, ensuring a more specific and sustained immune response against pathogens.

The antigen presentation capacity of monocytes is enhanced under inflammatory conditions and in response to certain stimuli. For instance, TLR7 stimulation has been shown to enhance the cross-presentation of cell-associated antigens by Ly6C+ classical monocytes [27]. In the context of cancer immunotherapy, antigen-loaded monocytes can induce potent therapeutic antitumor T cell responses [28]. These findings affirm monocytes’ integral role in maintaining immune surveillance and orchestrating adaptive immunity.

In addition to directly presenting antigens to lymphocytes, after migrating to tissues, monocytes differentiate into macrophages and dendritic cells, both of which are proficient antigen-presenting cells (APCs). Similar to monocytes, macrophages and dendritic cells engulf pathogens, process their proteins, and present antigenic peptides on their surface via major histocompatibility complex (MHC) molecules [29,30]. However, it is important to note that while monocytes can present antigens, their efficiency may be lower compared to specialized dendritic cells. The role of monocytes in antigen presentation appears to be particularly significant in inflammatory conditions and may serve as a complementary mechanism to dendritic cell-mediated antigen presentation.

### 2.3. Cytokine and Chemokine Production

In addition to their phagocytic and antigen-presenting capabilities, monocytes play an important role in cytokine and chemokine production, which is crucial for orchestrating immune responses. Upon activation, monocytes produce a wide array of cytokines, including tumor necrosis factor (TNF), interleukin-1 beta (IL-1β), IL-6, and IL-10, which modulate inflammation and immune cell recruitment. For instance, TNF and IL-1β are key pro-inflammatory cytokines that promote the activation and recruitment of other immune cells to sites of infection or injury [31]. IL-6 plays a dual role by supporting both pro-inflammatory and anti-inflammatory pathways, depending on the context of its release [32].

Chemokines are a family of small cytokines that activate G-protein-coupled receptors on monocytes. Chemokines produced by monocytes, such as monocyte chemoattractant protein-1 (CCL2/MCP-1), are essential for the recruitment of monocytes and other leukocytes to inflamed tissues. CCL2 binds to the chemokine receptor 2 (CCR2) on monocytes, facilitating their migration from the bone marrow to sites of inflammation [33]. Additionally, monocytes can produce CCL5 (RANTES) and CXCL10 (IP-10), which further enhance the recruitment of T cells and other immune cells, thereby amplifying the immune response [34].

The production of cytokines and chemokines by monocytes is tightly regulated and can be influenced by various factors, including pathogen-associated molecular patterns (PAMPs) and damage-associated molecular patterns (DAMPs). This regulatory mechanism ensures a balanced immune response, preventing excessive inflammation that could lead to tissue damage. Understanding the specific pathways and triggers for cytokine and chemokine production in monocytes is crucial for developing targeted therapies for inflammatory and infectious diseases [35].

### 2.4. Chemotaxis

Chemotaxis is a crucial effector function of monocytes, enabling their directed migration towards sites of infection or inflammation. This process is primarily mediated by MCP-1/CCL2, a key chemokine regulating monocyte migration and infiltration through its interaction with its receptor, CCR2 [36]. Upon activation, monocytes undergo a series of coordinated events involving the upregulation of adhesion molecules expression, cytoskeletal rearrangements, and polarization to facilitate their movement along chemokine gradients [37]. The chemotactic response involves the activation of intracellular signaling cascades, including the mobilization of intracellular calcium and the activation of kinases such as PI3K and MAPK [38]. These signaling events lead to the activation of integrins, which mediate firm adhesion to the endothelium, and the formation of specialized structures, like focal adhesions and the uropod, essential for directional movement [37]. Importantly, chemotaxis is not only crucial for monocyte recruitment to inflammatory sites but also plays a role in their normal physiological trafficking and immune surveillance [39]. Defects in monocyte chemotaxis have been associated with various pathological conditions, including acquired immune deficiency syndrome (AIDS), where impaired monocyte migration contributes to the compromised immune response [39] and Alzheimer’s disease [40,41]. Understanding the molecular mechanisms underlying monocyte chemotaxis is essential for developing targeted therapies for inflammatory disorders and improving our comprehension of the immune system’s functionality.

### 2.5. Tissue Repair

Monocytes play a role in tissue repair through their ability to differentiate into macrophages and their direct contributions to the repair process. Recent studies have elucidated the complex interplay between monocyte subsets and their functions in various stages of tissue repair. Classical CD14++CD16− monocytes recruited to sites of injury differentiate into inflammatory macrophages that promote debris clearance and initiate the repair cascade. These cells secrete pro-inflammatory cytokines and growth factors that stimulate angiogenesis and fibroblast activation. As the repair process progresses, there is a shift towards non-classical CD14+CD16++ monocytes, which can differentiate into reparative macrophages that support the resolution of inflammation, tissue regeneration and remodeling [42].

The plasticity of monocytes and monocyte-derived macrophages is a key feature in tissue repair, allowing them to adapt their phenotype in response to environmental cues. This plasticity is particularly evident in chronic inflammatory diseases, where the balance between pro-inflammatory and pro-resolving monocyte functions can influence disease progression and tissue repair outcomes [43]. Recent research has also highlighted the potential of biomaterial-based approaches to modulate monocyte behavior in tissue repair contexts, offering new avenues for therapeutic interventions [44].

It is important to note that while monocytes are generally beneficial for tissue repair, their dysregulation can contribute to fibrosis and impaired healing in certain conditions. The specific roles of monocyte subsets can vary depending on the tissue type and nature of the injury, underscoring the need for context-specific investigations to fully elucidate their functions in tissue repair processes [42].

### 2.6. ROS/RNS Production

Monocytes produce reactive oxygen species (ROS) and reactive nitrogen species (RNS) as part of their effector functions [45]. ROS, including superoxide anion (O_2_^−^) and hydrogen peroxide (H_2_O_2_), are generated through the activation of the NADPH oxidase complex [46,47]. To effectively produce ROS and RNS, monocytes undergo metabolic alterations that contribute to monocyte activation. A metabolic shift to aerobic glycolysis [48], enhanced activity of the pentose phosphate pathway, and alterations in the tricarboxylic acid (TCA) cycle help create a pro-oxidant environment [49]. ROS and RNS produce positive feedback on monocytes throughout this process to further propagate the pro-inflammatory system. Initial monocyte activation occurs in response to various stimuli, such as pathogen-associated molecular patterns (PAMPs) and cytokines [47]. Upon activation, NADPH oxidase transfers electrons from NADPH to oxygen, forming superoxide anions, which subsequently transform into hydrogen peroxide [50]. RNS, primarily nitric oxide (NO), are synthesized by the enzyme inducible nitric oxide synthase (iNOS), which is upregulated during inflammatory responses [51]. Nitric oxide can react with superoxide to form peroxynitrite (ONOO^−^), a potent oxidant with antimicrobial properties [52].

The production of ROS and RNS by monocytes contributes to the elimination of pathogens through oxidative and nitrosative stress, damaging microbial DNA, proteins, and lipids. This antimicrobial activity is essential for controlling infections, particularly in the early stages of the immune response. Moreover, ROS and RNS act as signaling molecules, modulating various immune functions such as cytokine production, cell proliferation, and apoptosis [53]. However, excessive or uncontrolled production of ROS and RNS can lead to tissue damage and contribute to the pathogenesis of inflammatory diseases, including atherosclerosis [54], rheumatoid arthritis [55], Alzheimer’s disease [14,56] and sepsis [57].

In conclusion, the production of ROS and RNS by monocytes is a critical effector function that mediates pathogen clearance and regulates immune responses. Understanding the balance between their beneficial and detrimental effects is essential for developing therapeutic strategies to modulate immune responses in various diseases.

## 3. Effect of CMV on Monocyte Effector Functions

### 3.1. CMV–Monocyte Interactions

**Figure 2:**
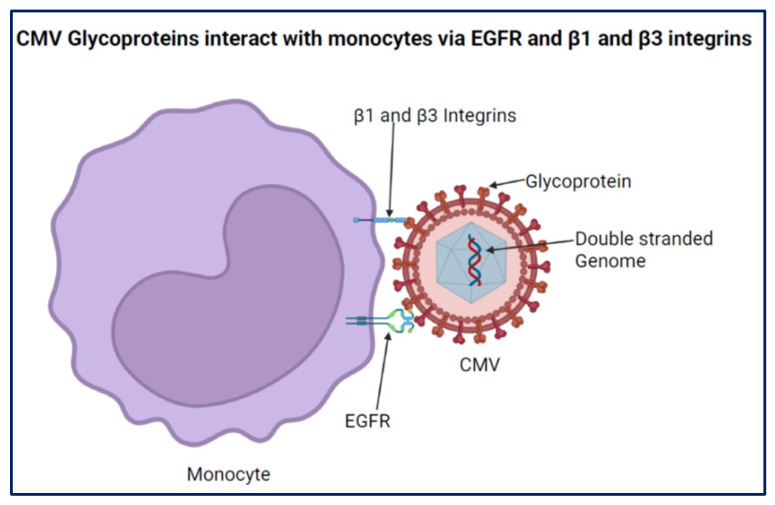
CMV interacts with monocytes via β1 and β3 integrins and EGFR.

CMV utilizes receptor-initiated signal transduction in monocytes to shape the biology of these cells (Figure 2) [58]. Indeed, the engagement of EGFR on monocytes by CMV glycoproteins is required for CMV entry and has been shown to mediate trans-endothelial migration of these cells [59]. In addition, EGFR and β1 and β2 integrin engagement are necessary for monocyte motility following CMV infection [60]. It is not known if one or all these interactions are responsible for modulating additional CMV-regulated monocyte effector functions.

### 3.2. Effect of CMV on Phagocytosis

**Figure 3:**
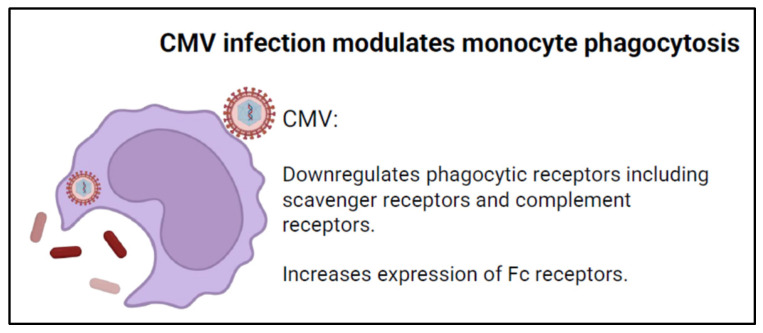
CMV infection modulates monocyte phagocytic function.

CMV infection has been shown to modulate the phagocytic ability of monocytes (Figure 3) which may compromise the immune system’s ability to handle infection. One mechanism by which CMV impacts phagocytosis is through a paradoxical effect of classical (CD14++, CD16−) monocytes. RNA sequencing of monocytes post-CMV infection demonstrates the upregulation of viral pathogen recognition receptors (PRRs) while also demonstrating the downregulation of pathogen associated molecular pattern (PAMP)-related scavenger receptors. The specific scavenger receptors impacted include CD36, which is responsible for fungal pathogen recognition [61]. The decreased expression of key phagocytotic receptors for fungi leads to a marked decreased ability to phagocytose key fungal pathogens, including *C. albicans* and *C. neoformans*, which are major contributors to morbidity in immunocompromised hosts [61,62]. These findings provide a molecular explanation for the increased susceptibility to fungal infections during or following infection with CMV. It remains unclear whether low level CMV reactivation associated with many types of acute illnesses is similarly involved in the risks for nosocomial fungal superinfections [63].

Other studies have demonstrated that complement receptors 3 and 4, CR3 and CR4, two important modulators of fungal and bacterial phagocytosis, are downregulated in both THP-1 cell line-derived macrophages and monocyte-derived human macrophages infected with CMV [64].

While published evidence demonstrates that CMV directly downregulates phagocytosis through scavenger and complement receptors, the effect of CMV infection on Fcγ-receptor-mediated phagocytosis is not fully clear. There is evidence that CMV may modify Fcγ receptor-mediated phagocytosis through the expression of cmvIL-10, a homologous cytokine to human interleukin-10 (hIL-10) [65] produced by CMV infected cells [66,67], thereby altering monocyte gene expression in an autocrine or paracrine fashion. Monocytes cultured with cmvIL-10 demonstrated increased expression of Fcγ receptors CD32 and CD64 [65]. CmvIL-10 increased Fcγ-receptor-mediated phagocytosis by monocytes similar to hIL-10 [65]. The role of enhanced Fcγ-mediated phagocytosis in monocytes in the pathogenesis of CMV disease is not clear, but it may play a role in viral dissemination. Similarly, CMV infection induces the expression of viral FcγRs, such as gp34 and gp68, which can bind to the Fc region of immunoglobulins and inhibit the activation of host FcγRs (FcγRI, FcγRIIA, and FcγRIIIA) on monocytes and other immune cells [68]. This prevents IgG-mediated triggering from activating host FcγRs, thereby impairing antibody-dependent cellular phagocytosis (ADCP) and other Fc-mediated effector functions [68]. Further studies on the impacts of CMV infection on Fcγ-mediated phagocytosis are necessary to clarify the biologic significance and implications of altering this phagocytic pathway on CMV disease and the innate immune response.

### 3.3. Effect of CMV on Antigen Presentation

**Figure 4:**
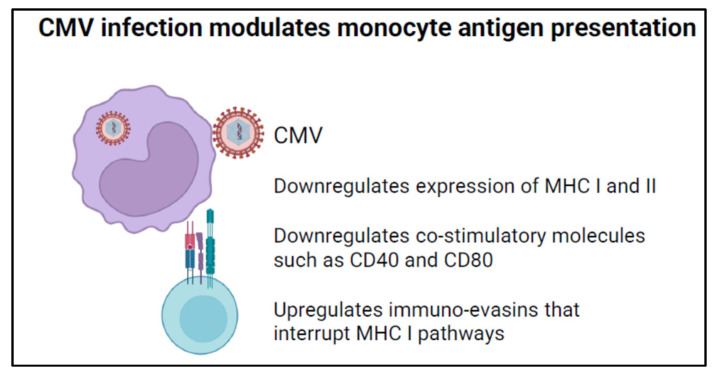
CMV modulates antigen presentation by monocytes.

CMV infection affects the antigen-presenting function of monocytes through multiple mechanisms (Figure 4). First, CMV decreases the effectiveness of antigen presentation by inhibiting the differentiation of monocytes into dendritic cells (DCs) [69,70,71]. DCs are the major antigen-presenting cells and play a key role in continuing immune activation via signaling to T cells [72]. By blocking differentiation-inducing cytokines like IL-4 and GM-CSF, CMV prevents monocyte differentiation into CD1a-positive dendritic cells [69]. Monocytes are less efficient at antigen presentation than dendritic cells. Thus, inhibiting monocyte differentiation decreases antigen presentation, making the host more susceptible to infection.

Second, in the setting of acute infection, CMV downregulates the expression of major histocompatibility complex (MHC) class I and II molecules [70]. Interestingly, cmvIL-10 inhibits cell surface expression of MHC I and II in stimulated monocytes in contrast to its effects on Fc-mediated phagocytosis [67]. CMV can also downregulate the co-stimulatory molecules CD40 and CD80 on the surface of monocytes and immature DCs [70,71]. Downregulation of these co-stimulatory molecules may inhibit antigen loading of existing MHC molecules. Third, CMV expresses immune evasins, glycoproteins that interrupt classical MHC class I pathways [73]. Indeed, CMV-encoded proteins, US3, US6, and US10, can retain MHC class I molecules in the ER, inhibit peptide transport by the transporter associated with antigen processing (TAP), or delay trafficking of MHC I to the cell surface [74,75,76,77,78]. The combination of these effects further undermines CD4+ and CD8+ T cell immunity and may help CMV go unchecked in the settings of acute infection and reactivation. 

### 3.4. Effect of CMV on Cytokine Production

**Figure 5:**
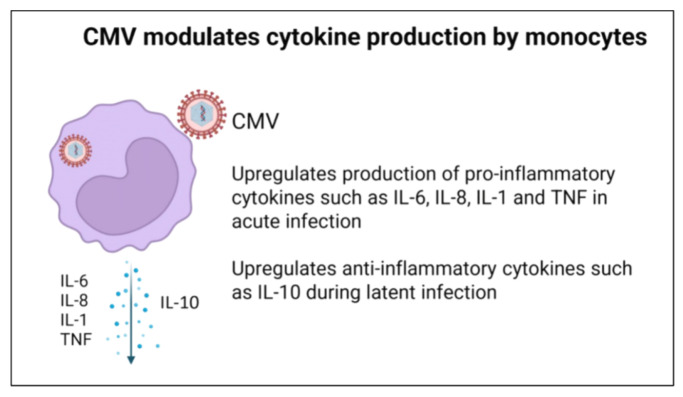
CMV modulates monocyte cytokine production.

Cytokines are crucial players in regulating immune responses and inflammation. Monocytes infected with CMV produce a mixed phenotype of pro- and anti-inflammatory cytokines (Figure 5). This may reflect incomplete infection of the cell population as well as various states of CMV infection. The resulting immune environment promotes pathways beneficial for viral persistence while allowing cellular CMV to evade the immune system. Pro-inflammatory cytokines IL-6 and IL-8 are increased when monocytes are incubated with UV-inactivated CMV virus, indicating cytokine production results from cellular contact with viral particles rather than new gene expression [79]. Increased cytokine signaling requires the pattern recognition receptors TLR2 and CD14. It is possible that such receptors may be responsible for recognizing CMV virions (in addition to EGFR and β1 and β3 integrins) leading to inflammatory signaling, though such an interaction remains to be determined [79]. Other pro-inflammatory cytokines including TNF are also increased through the activation of p38 kinase and NF-kB signaling pathways in monocytes [80]; this requires further investigation as some reports show decreased TNF production with acute CMV infection or reactivation [71].

In addition to increasing cytokine gene expression, further mechanisms are involved in the CMV-modified monocytic cytokine production. The binding of CMV to monocytes, and the major CMV glycoproteins gB (UL55)- and gH (UL75)-mediated signaling, upregulates the secretion of IL-1β. This expression required the activation of NF-kB, a transcription factor responsible for regulating IL-1β gene expression [81]. Although more research is needed, the CMV-mediated increase in IL-1β expression could be linked to chronic inflammation associated with CMV infections and further complicate the analysis of CMV infection in immunocompromised hosts.

While increased pro-inflammatory cytokines may be crucial to acute infection and CMV-associated inflammatory diseases, CMV also modulates anti-inflammatory cytokines for immune evasion and latency. For example, CMV upregulates the production of IL-10, an anti-inflammatory cytokine shown to prevent the differentiation of monocytes into dendritic cells [69,82]. In support of this anti-inflammatory effect, recombinant cmvIL-10 inhibits IFN-γ, IL-1α, IL-6, GM-CSF, and TNF production by preventing NF-κB signaling in monocytes [67]. Furthermore, monocytes and DCs infected with CMV have a decreased production of interleukin-12, a key cytokine in Th1-mediated immunity. Decreased cytokine signaling presents another barrier to T cell activation and the cytotoxic killing of CMV.

In addition to utilizing viral proteins for downregulating cytokine production, CMV utilizes microRNA (miRNA) to decrease the expression of pro-inflammatory cytokines during latency and later infection. Through these miRNA species, CMV downregulates IKKa and IKKB signaling factors, limiting the production of pro-inflammatory cytokines IL-6 and TNF-a to remain dormant and evade host immunity [83]. It is possible that cmvIL-10 and the effect of CMV generated miRNA can help spread the effects of CMV to non-infected monocytes. Thus, these modulations likely propagate the effects of CMV infection on the entire monocyte population and amplify such effect(s). 

### 3.5. Effect of CMV on Chemotaxis

CMV-infected monocytes exhibit decreased chemokine receptor expression, which can impair migration to sites of infection or injury. These downregulated chemokine receptors include CCR1, CCR2, CCR5, and CXCR4. Such downregulation occurs even in the absence of viral gene expression and is believed to be due to increased internalization of these receptors, rather than decreased gene expression or protein degradation [84]. Functionally, CMV-infected monocytes were unable to migrate toward CCL2, CCL5, CXCl12, CCL19, or CX3CL1, key chemokine modulators of adaptive immunity critical for monocyte migration and recruitment to sites of inflammation, thereby potentially compromising the host’s ability to mount an effective immune response.

**Figure 6:**
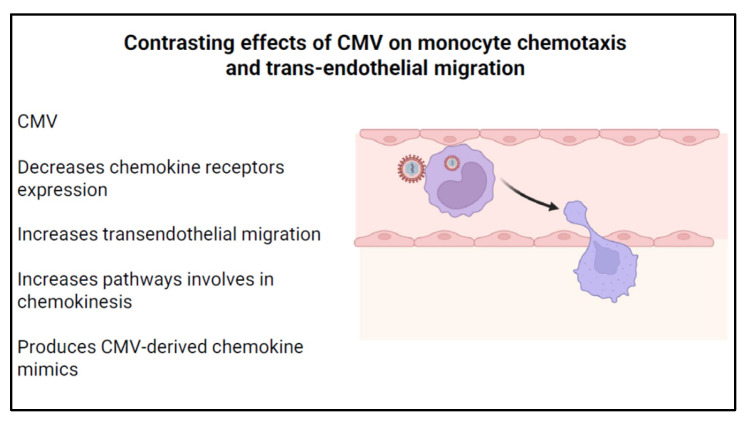
CMV modulates monocyte migration and chemotaxis.

In contrast to downregulated chemokine-mediated migration, CMV-infected monocytes demonstrate increased trans-endothelial migration. This increased migration is independent of viral gene expression, and occurs in monocytes incubated with both live CMV and UV-inactivated CMV strains [85]. Several mechanisms are thought to be responsible for such an increase. First, cell motility is increased [85]. Second, increased monocyte adhesion to endothelial cells, a key step in both early and late stages of trans-endothelial migration, is also increased. This is due to enhanced expression of β1 integrin, occludin, and ZO-1, proteins involved in monocyte adhesion and diapedesis [85]. Additional studies also suggest that CMV-infection of the endothelial cells, in turn, increases the migration of monocytes (Figure 6) [86]. 

It has been speculated that CMV could also impact migration through chemokinesis, a motility process independent of chemokines [87]. Chemokinesis is dependent on PI(3)K and the actin cytoskeleton and requires activation and signaling via integrin and EGFR pathways. These pathways are increased as a result of CMV infection [1]. These secondary pathways allow CMV-infected monocytes to migrate more effectively, even without the presence of chemotactic signaling. Downregulating chemotaxis-dependent migration while maintaining chemotactic-independent migration may help CMV evade the adaptive immune system while using it for further dissemination.

CMV can also influence chemotaxis and migration through the production of chemokine-mimics encoded in the CMV genome. Chemokine homologs are common to many viruses and provide a method of immune dissemination in acute infection. In murine CMV, viral chemokines that show similarity to host CC chemokines, MCK-1 and MCK-2, were crucial to viral dissemination through monocytes [88]. Deletion of the MCK-1/MCK-2 gene resulted in decreased viremia and the failure of murine CMV dissemination to salivary glands, a common reservoir of CMV. In human CMV, UL128, a viral protein with functional similarities to human CC chemokines, promoted the recruitment and proliferation of peripheral blood mononuclear cells [89]. Lastly, chemokine receptor homologs, like US28, may also promote monocyte chemotaxis through the activation of G-protein signaling cascades [90,91].

Based on these observations, it is clear that CMV can regulate the migration of monocytes. Decreased chemokine-mediated monocyte migration can help CMV evade the immune response while increasing viral dissemination via increased CMV-induced monocyte chemokinesis and trans-endothelial migration and in response to CMV-induced chemokine mimics. Understanding the stages of CMV infection that regulate each of these migratory functions will clarify the role of this important monocyte effector function in CMV pathogenesis.

### 3.6. Effect of CMV on Tissue Repair and Immune Regulation

CMV infection can have a significant impact on tissue repair. CMV-infected monocytes have reduced phagocytotic abilities, altered migration, and improper signaling of T cells. Dysregulation of these typical functions leads to improper tissue repair [92]. In contrast, prolonged CMV infection can lead to a chronic inflammatory state caused by cytokines from CMV-infected monocytes. High levels of IL-6, IL-8, and IL-1β create a cellular environment that is less favorable to tissue regeneration [81]. Adhesion to endothelial cells by CMV-infected monocytes may also contribute to excessive inflammation [93]. We propose that these apparent contrasting effects on the inflammatory process appear to have additive negative effects on tissue repair.

While the transition from monocytes to macrophages is upregulated through interactions with CMV, there is an altered differentiation into CD1a-positive dendritic cells by IL-4 and GM-CSF [69]. Dendritic cells differentiated from CMV-infected monocytes demonstrate an inability to secrete IL-12 in response to LPS stimulation, decreased phagocytosis, and decreased induction of TH-1 cell differentiation. Although only a subset of monocytes was infected with CMV, there was widespread disruption of GM-CSF signaling in the entire population of dendritic cells. These disruptions were due to an inhibition of STAT5 activation caused by paracrine signaling of soluble factors secreted by CMV-infected monocytes [94]. Because CMV-infected dendritic cells exhibit decreased differentiation, we propose that their role in tissue repair is also dysregulated.

### 3.7. Effect of CMV on ROS Production

An important component of the monocytes’ host defense mechanisms is the generation of reactive oxygen species (ROS) and reactive nitrogen species (RNS). ROS include superoxide (O_2_^−^), hydrogen peroxide (H_2_O_2_), and hydroxyl radical (OH^−^) [95]. Within monocytes, engulfed material enters the phagolysosome where it encounters superoxide, proteases, and other molecules that can kill microbes. ROS are secreted extracellularly, where they can perform their host defense functions [96]. This effector function of monocytes is important in the host’s response to bacterial, fungal and viral pathogens [97]. ROS also play a major role as cellular signaling molecules during inflammation [98].

During acute infection or reactivation, CMV upregulates ROS generation even in the absence of DNA replication within monocytes. This modulation is thought to be associated with increased transduction of signaling cascades of ROS generation or through the augmentation of cytosolic factors involved in ROS generation [99]. Similarly, when THP-1 cells, a human monocytic cell line, were infected with CMV, there was a significant increase in ROS generation during active CMV infection [100]. Therefore, in contrast to the role of ROS in bacterial and fungal killing, the increased levels of ROS are beneficial to the establishment of CMV within the host. Increased ROS production enhances CMV gene expression, which helps promote viral establishment during acute infections [101]. Additionally, oxidative stress and inflammatory responses to increased ROS may create a cellular environment more favorable to viral replication [102].

In contrast to the upregulation of ROS signaling and production observed with active CMV replication in monocytes, CMV evades the immune response during latency by downregulating ROS production by macrophages. This suppressive effect on ROS production during latency is believed to be regulated by a long non-coding RNA (lncRNA) called β2.7. CMV variants without the β2.7 gene were less effective at establishing latency within host monocytes and suppressing ROS production [103]. These findings suggest that the ability to downregulate ROS production in the host allows CMV to remain latent and undetected by the host immune system. CMV’s dual effect on monocyte and macrophage ROS production links this important monocyte/macrophage effector function to two important paths involved in CMV pathogenesis in immunocompromised hosts: one path leading to replication and one leading to the establishment of life-long latency within myeloid cells.

## 4. Clinical Implications

The effects of cytomegalovirus (CMV) infection on monocyte effector functions have significant clinical implications, particularly in the immunosuppressed host. Solid organ transplant (SOT) patients, those with uncontrolled HIV/AIDS, and those with iatrogenic immunosuppression are at high risk of complications of CMV-related illness. Although CMV possesses many mechanisms to disrupt host immunity, the modulation of monocyte effector functions produces a paradoxical change to innate immune responses, leading to both immunosuppression (e.g., opportunistic infection) and immune stimulation (e.g., graft rejection). These dual and paradoxically opposed effects provide insight into the importance of CMV infection in active, reactivated, and latent states.

### 4.1. Phagocytosis

A crucial effector function in host defense against pathogens. Monocytes are responsible for phagocytosis, with the disruption of this ability with CMV infection being important in the increased susceptibility to infection. The reduced phagocytic ability of monocytes is detrimental against clinically important and common yeasts including *Candida albicans* and *Cryptococcus neoformans*. This is associated with decreased expression of key pattern recognition receptors such as CD36, MRC1, and complement receptor 3 [61]. A higher risk of invasive fungal infections is amplified in immunocompromised individuals with decreased immunity against opportunistic fungi.

Increased infections with fungal pathogens create an issue in post-SOT management. The post-transplant regime is complex with a variety of immunosuppressive, antibacterial, antiviral, and antifungal agents [104]. Impaired phagocytosis of *C. albicans* and *C. neoformans*, two of the three most common fungal infections post-transplant [105], may necessitate more aggressive antifungal prophylaxis in CMV-infected transplant recipients. These fungal infections in patients following SOT may increase the risk for allograft rejection (possibly associated with decreases in immunosuppression) and have a 12-month mortality of 34% and 27%, respectively [105]. Strategies to restore phagocytic function in CMV-infected monocytes could potentially mitigate the indirect effects of CMV in transplantation.

### 4.2. Antigen Presentation

Decreased antigen presentation by monocytes in patients with CMV may have significant clinical implications. First, decreased antigen presentation will reduce the stimulation of T cell responses against both CMV and other opportunistic pathogens. CMV-infected monocytes may also exhibit increased expression of regulatory co-stimulatory molecules including CD86, potentially enhancing T cell activation in certain contexts [69]. The expression of CD86 without proper antigen presentation may result in T cell anergy, increasing the risk for opportunistic (viral and fungal) infections.

### 4.3. Production of Pro-Inflammatory Cytokines

A major impact on monocyte effector function is the production of pro-inflammatory cytokines during acute infection and with reactivation of CMV. The upregulation of key cytokines, IL-6, IL-1β, and TNF-α, creates a pro-inflammatory environment that is significant in both acute and chronic diseases. Of major concern is that heightened levels of inflammation in SOT patients may contribute to allograft dysfunction, rejection, and graft-versus-host disease in transplant recipients.

CMV is also associated with important organ-specific infections in immunocompromised hosts including CMV colitis, retinitis, hepatitis, pneumonitis, and encephalitis. There is evidence that the pro-inflammatory cellular environment may contribute to chronic inflammatory diseases including atherogenesis, accelerated atherosclerosis in cardiac transplant recipients, and chronic lung allograft dysfunction (CLAD) in lung transplant recipients [106].

### 4.4. Implications for Therapeutics

The impact of CMV on monocyte differentiation and function has implications on the development of therapeutic strategies. The targeting of pathways involved in monocyte differentiation and cytokine production could mitigate the impacts of CMV infection and improve clinical outcomes for affected patients. For example, therapies that inhibit the differentiation of monocytes into anti-inflammatory macrophages or enhance the phagocytic capacity of monocytes could reduce viral persistence and improve viral immune clearance [107].

In conclusion, the complex modulation of monocyte effector functions by CMV has far-reaching clinical implications in transplantation. A nuanced understanding of these effects is crucial for optimizing management strategies, including antiviral therapy, immunosuppression, and the development of cellular therapies, to improve outcomes in CMV-infected transplant recipients. The immunosuppressive effects of CMV on monocytes may provide a mechanism to downregulate adaptive immune responses that would otherwise lead to clearance, thereby promoting viral persistence.

Conversely, CMV infection also induces pro-inflammatory and immunostimulatory responses in monocytes. This includes the upregulation of viral pattern recognition receptors, inflammasome components (e.g., AIM2, IFI16), pro-inflammatory mediators associated with allograft rejection and graft-versus-host disease [108]. This could also lead to end-organ inflammatory-mediated diseases occurring during primary infection and reactivation, where CMV viral replication is the highest.

These divergent effects of CMV on monocyte function may be host and time dependent and suggest that the pro-inflammatory phenotype may require tailored immunosuppression strategies to mitigate the risk of allograft rejection. The heterogeneity in monocyte responses to CMV infection, as revealed by single-cell transcriptomics, suggests that personalized approaches may be needed to address the variable effects in individual patients. Understanding these CMV-mediated alterations in monocyte function opens avenues for targeted therapies. Strategies to restore phagocytic function or modulate inflammatory responses in CMV-infected monocytes could potentially mitigate the indirect effects of CMV in transplantation. Additionally, the immunostimulatory properties of CMV immunoglobulin (CMVIG) on innate immune cells, including monocytes, may be harnessed to enhance anti-CMV immunity in transplant recipients [109].

## 5. Conclusions

CMV infection has a profound impact on monocyte effector functions, impairing phagocytosis, antigen presentation, cytokine production, cell migration to sites of infection, and differentiation into effective immune cells. These alterations contribute to immune compromise and to susceptibility to infections, heightened inflammation, graft rejection, and long-term complications in immunocompromised individuals. Understanding the mechanisms by which CMV modulates monocyte functions is crucial for developing targeted therapies to mitigate the effects of CMV infection and improve clinical outcomes for affected patients.
